# Criminal Germans and British Prisoners

**Published:** 1916-04-15

**Authors:** 


### CRIMINAL GERMANS AND BRITISH PRISONERS.

### A TERRIBLE STORY OF BRUTAL CRUELTY.

### Heroic British Practitioners.

One of the most terrible stories of the war is contained in the Report by the Government Committee on the Treatment by the Enemy of British Prisoners of War, upon the conditions of the camp at Wittenberg during and before the epidemic of typhus which devastated it in the first six months of 1915. The following digest of the Report is based on *The Times* summary:—

It is believed that before and during the progress of the typhus there were at least 15,000 prisoners in the camp, and there may have been as many as 16,000 or 17,000—an enormous population for so restricted an area as 10½ acres. The winter of 1914-15 was extremely severe and the cold at Wittenberg intense, but the heating arrangements for the camp were altogether inadequate. Often there was no coal for the stoves, and the temperature was so low that the men had always to keep every window shut to husband what little warmth there was. This greatly aggravated the evil of the overcrowding.

#### Want of Food and Clothing.

Moreover, the men were insufficiently clothed. From most of the British prisoners their overcoats were taken on the day of their capture; none were given them in exchange. Their remaining clothes were often in rags; some of the men had even to use their blankets as clothing. Occasionally a prisoner had received a thin cotton shirt, but there were many with neither boots nor socks; many others had their feet wrapped in straw.

The food with which they were supplied was bad and insufficient. After making every allowance, the committee are left with a record of suffering owing to the lack of wholesome food which they cannot but deplore. And the spread of typhus, when it came, was much facilitated by a camp regulation, not confined to Wittenberg, which enjoined that the prisoners of all nationalities should be mixed together. Normally, there was only one mattress for every three prisoners, and every British prisoner was compelled to have one French and one Russian prisoner to share his mattress with him.

Now typhus, as was fully recognised by the Russian doctors themselves, was unfortunately to some extent—but through no fault of their own—latent amongst some of the Russian troops, and it is a well-known medical fact that lice are the great carriers of that disease, while it is, of course, notorious that the men of all armies in the field, including the British, are plagued with lice. As a protection against typhus, therefore, the separation of the infected was an elementary precaution. But at Wittenberg no adequate measures were taken even to free the prisoners, on their arrival at the camp, from the lice. The only provision for personal cleanliness there made for the men was one cupful of soft soap issued at intervals of many weeks to a room containing at least 120. In consequence, the men became increasingly verminous, and that condition, coupled with the cold and want of proper nourishment, was undoubtedly the principal inducing cause of the epidemic which supervened.

The medical and surgical arrangements were under the charge of Oberstabsarzt Der. Aschenbach and his German assistants. The epidemic broke out in December 1914. Thereupon the German staff, military and medical, precipitately left the camp, and thenceforth until the month of August 1915, with the exceptions detailed later on, no communication was held between the prisoners and their guards except by means of directions shouted from the guards or officers remaining outside the wire entanglements of the camp. All supplies for the men were pushed into the camp over chutes. The food for the hospital and medical officers was passed in on a trolly over about 20 yards of rail, worked by winches at either end so as to avoid all contact between the prisoners and the outside world. No medical attention during the whole time was provided by the German staff.

From the month of November 1914, thirteen English doctors had been detained at Halle. They were none of them required for attendance upon their own men, and it is difficult to understand how, consistently with the Geneva Convention, their continued detention was justifiable. Indeed, in direct defiance of the provisions of that Convention, these doctors were treated as ordinary prisoners of war, and the Committee cannot resist the suspicion that they were deliberately detained by the German authorities so that they might be made available, if need be, for work of danger in relief of their own staff. Be that as it may, after three months' wrongful detention these doctors were, on February 10, 1915, informed that they were to be distributed amongst the other German camps, and particularly that six were required for the camp at Wittenberg. By arrangement amongst themselves the six sent there were Major Fry, Major Priestley, Captain Sutcliffe, Captain Field, Captain Vidal, and Captain—then Lieutenant—Lauder. No reason was given for the order that they should go to Wittenberg, and it was from the guard on the train that they first heard of typhus there.

On arrival at Wittenberg they were marched to the camp. They visited the different compounds. They were received in apathetic silence. The rooms were unlighted, the men were aimlessly marching up and down; some were lying on the floor probably sickening for typhus.

Of four officers, Captain Lauder alone survives, and the conditions as he describes them during the period between February 11 and March 7 are full of horror. The wonder is that any prisoner escaped infection. Captain Lauder found that in the improvised hospital there were no mattresses at all. This, of course, was known throughout the camp, and in consequence there were many typhus patients scattered over the compounds who were determined not to come into the hospital if they could help it. In one compound alone Captain Lauder discovered fifty hidden cases of typhus. Further, when a patient was brought from the compound to the hospital either the mattress on which he had lain was brought with him or it was left behind in his bungalow. If it was brought with him his former companions were left without anything to sleep on; if it was left behind his still uninfected companions were left to sleep upon the infected mattress, and it was almost inevitable that they should catch the disease. Again, in the absence of stretchers, all the typhus cases had to be carried down to the hospital on the tables on which the men ate their food, and there was no possibility of washing these tables, because there was practically no soap in the camp. Moreover, the German authorities at first refused to allow the whole of Compound No. 8 to be used for typhus patients. They required that these should be mixed with other sufferers, a regulation for which it seems impossible to suggest any justification. The result simply was to spread the infection to those already afflicted in some other way.

During the first month the food ration for each patient was half a *petit pain* and half a cup of milk each per day. The only soup to be got was from the camp kitchen, but that came up in a wooden tub without a cover, and it arrived at the hospital—so one of the prisoners says—full of dust and dirt. It was hopeless diet for patients in a fever.

The camp conditions were too much for each of the four medical officers who were left there; three of them, Major Fry, Captain Sutcliffe, and Captain Field, were attacked by the disease and died. There is no doubt in the minds of the Committee that the conditions to which the camp authorities had reduced the camp and the prisoners they had abandoned was directly responsible for the deaths of these devoted men. Lieutenant Lauder was finally stricken with the disease on March 7. He alone of the officers attacked finally recovered. When convalescent he bravely resumed his duty.

On March 7 Major Priestley and Captain Vidal were directed to return to the main camp. There were then about 1,000 cases of typhus in the camp, and fresh cases were coming in at the rate of about fifty, and sometimes more, a day. There were at that time about 150 British cases. The British sick were lying scattered amongst the French and the Russians, lying sometimes dressed in French, Belgian, or Russian uniforms, which made them difficult to recognise. Major Priestley saw delirious men waving arms brown to the elbow with fæcal matter. The patients were alive with vermin; in the half light he attempted to brush what he took to be an accumulation of dust from the folds of a patient's clothes, and he discovered it to be a moving mass of lice.

### Post Typhus Gangrene.

The difficulty in the way of obtaining sufficient drugs and dressings was for a long time extreme. Camphorated oil, Captain Lauder says, could never at Wittenberg, contrary to his experience in other German camps, be secured in adequate quantity, yet this was practically the only stimulant available. Day after day a list of medical requisites would be sent out and only a third of the things requested would be supplied. Bed sores were common. In several cases toes or whole feet became gangrenous, and sufficient bandages were not available to dress them. One of the patients now returned to this country, Private Lutwyche, of the 1st Battalion Royal Scots Fusiliers, had in May to have one leg amputated below the knee, and in July the other leg amputated at the same place, in both cases owing to gangrene. Had dressings at the proper time been available both feet would in all probability have been saved. And his case does not stand alone.

On one occasion only during the whole course of the epidemic did Dr. Aschenbach enter the hospital or even the camp. His visit took place about four weeks after Major Priestley's arrival and after some kind of order had been evolved. He came attired in a complete suit of protective clothing, including a mask and rubber gloves. His inspection was brief and rapid. For his services in combating the epidemic Dr. Aschenbach, the Committee understand, has been awarded the Iron Cross.

The dead were buried in a cemetery formed out of a part of the camp. The Germans sent in a certain number of coffins every day into which the bodies of the dead were put and carried out by their comrades through a gate in the barbed wire. There was not sufficient room for burial of so many and the coffins were piled one upon another, but the Committee do not think there was any special danger in the arrangement. What the prisoners found hardest to bear in this matter were the jeers with which the coffins were frequently greeted by the inhabitants of Wittenberg, who stood outside the wire and were permitted to insult their dead.

During the first two months the typhus was hæmorrhagic typhus; it was of a milder type later on. There were between 250 and 300 English cases and there were 60 deaths amongst them. In the earlier days it was often necessary to discharge the patients from hospital before they were fit to be removed. In many cases these men had to go back to their barrack-room and lie on the bare floor, as no fresh beds or mattresses were provided for a long time.

Captain Vidal says that the conditions were thoroughly realised by the German authorities without any effort being made by them to bring about an improvement.

After the middle of April, however, beds and clothing were, as above appears, gradually obtained for the hospital, and as the weather became warmer the cases rapidly decreased in number. With the decrease in the patients, the supplies became adequate, so that now every patient in the

Wittenberg Hospital, whatever be his ailment, has a bed and proper hospital clothing.

Incredible as it may seem, the action of the officers and guards in precipitately deserting the camp and thenceforth controlling its caged inmates with loaded rifles from the outside was only in keeping with the methods and conduct of these men throughout. The cruelty of the administration at Wittenberg Camp from the very commencement has become notorious. Savage dogs were habitually employed to terrorise the prisoners; flogging with a rubber whip was frequent; men were struck with little or no provocation, and were tied to posts with their arms above their heads for hours.

### "Schweine Englaender."

And the callousness during the outbreak even of so prominent an officer as Dr. Aschenbach is illustrated by an incident related by Captain Lauder. Shortly after their arrival at the camp Major Fry, with Captain Lauder, was begging Dr. Aschenbach, standing outside the entanglements, for some medical requisite urgently required. One of his staff with Dr. Aschenbach was apparently favourably inclined towards the request, but it was curtly refused by Dr. Aschenbach, who turned away with the words, "Schweine Englaender." To the Committee an incident like that, with all that it implies, speaks volumes. The Committee draw attention to the splendid work of the British medical staff and orderlies during the epidemic. Major Priestley's work has already been referred to in this report; it was beyond all praise. Captain Vidal was, in the words of one of the prisoners, the idol of the camp; and Major Priestley says of Captain Lauder that he cannot sufficiently express his admiration for his pluck and skill and for the unobtrusive way in which he did his duty. It was he who, at the beginning, bore the brunt of the outbreak.

All these officers concur in praising the splendid bearing of the orderlies. They each of them volunteered for the work; they tended prisoners of all nationalities. They all of them with full understanding, for they were all warned, risked their lives without a thought, and many of them died at their post. The Committee hope to be able in due course to supply His Majesty's Government with a full list of these heroic souls.

